# The Picture Talk Project: Starting a Conversation with Community Leaders on Research with Remote Aboriginal Communities of Australia

**DOI:** 10.1186/s12910-017-0191-z

**Published:** 2017-05-11

**Authors:** E.F.M. Fitzpatrick, G. Macdonald, A.L.C. Martiniuk, H. D’Antoine, J. Oscar, M. Carter, T. Lawford, E.J. Elliott

**Affiliations:** 1 Discipline of Paediatrics and Child Health, Sydney Medical School, University of Sydney, Sydney, NSW Australia; 2 The Sydney Children’s Hospital Network, 4 Governor Phillip Place, West Pennant Hills, Sydney, 2125 NSW Australia; 3 Department of Anthropology, University of Sydney, Sydney, NSW Australia; 4 Sydney Medical School, University of Sydney, Sydney, NSW Australia; 5 The George Institute for Global Health, Sydney, NSW Australia; 6 Dalla Lana School of Public Health, University of Toronto, Toronto, Canada; 7 Menzies School of Health Research, Darwin, NT Australia; 8 Marninwarntikura Women’s Resource Centre, Fitzroy Crossing, WA Australia; 9 Nulungu Research Institute, The University of Notre Dame, Broome, Australia; 10 Nindilingarri Cultural Health Services, Fitzroy Crossing, WA Australia; 11 Kimberley Aboriginal Law and Culture Centre, Fitzroy Crossing, Australia

**Keywords:** Australian aboriginal people, Indigenous, Consent, Engagement, Research, Community

## Abstract

**Background:**

Researchers are required to seek consent from Indigenous communities prior to conducting research but there is inadequate information about how Indigenous people understand and become fully engaged with this consent process. Few studies evaluate the preference or understanding of the consent process for research with Indigenous populations. Lack of informed consent can impact on research findings.

**Methods:**

The Picture Talk Project was initiated with senior Aboriginal leaders of the Fitzroy Valley community situated in the far north of Western Australia. Aboriginal people were interviewed about their understanding and experiences of research and consent processes. Transcripts were analysed using NVivo10 software with an integrated method of inductive and deductive coding and based in grounded theory. Local Aboriginal interpreters validated coding. Major themes were defined and supporting quotes sourced.

**Results:**

Interviews with Aboriginal leaders (*n* = 20) were facilitated by a local Aboriginal Community Navigator who could interpret if necessary and provide cultural guidance. Participants were from all four major local language groups of the Fitzroy Valley; aged 31 years and above; and half were male. Themes emerging from these discussions included Research—finding knowledge; Being respectful of Aboriginal people, Working on country, and Being flexible with time; Working together with good communication; Reciprocity—two-way learning; and Reaching consent.

**Conclusion:**

The project revealed how much more there is to be learned about how research with remote Aboriginal communities should be conducted such that it is both culturally respectful and, importantly, meaningful for participants. We identify important elements in community consultation about research and seeking consent.

**Electronic supplementary material:**

The online version of this article (doi:10.1186/s12910-017-0191-z) contains supplementary material, which is available to authorized users.

## Background

If the process of community engagement and seeking consent for research is not conducted a way that is understood or respectful of Aboriginal cultural protocol this can have significant impact on participation rates, community and researcher relationships and may even effect research findings, not to mention long term relationships with Aboriginal communities in Australia. There is a need for research that is prioritised, led and clearly understood by Indigenous communities if we are to identify and address disparities in health and wellbeing. In this paper the term ‘Indigenous’ will be used when referring to any population which is considered to be Indigenous [1, 2]. When appropriate we will use a specific name such as ‘Australian Aboriginal’ or ‘Torres Strait Islander’. Our recent systematic review identified research that describes in detail, or evaluates, the understanding and preference for the way information is presented while seeking consent for research with Indigenous populations [3]. Few publications in the international literature report on the consent process with Indigenous people [3–5]. Even less frequently do they contain any evaluation of preference for the methods used to communicate information about research or participants’ understanding of the information provided during the consent-seeking process [3–5]. Miscommunication between researchers and Indigenous communities continues to be reported [6, 7] and this may foster mistrust of researchers despite their good intentions and lead to poor research outcomes.

Current guidelines highlight important key values and principles for conducting research with Indigenous populations [3, 4, 8–13]. For example, the National Health and Medical Research Council’s guideline, ‘*Keeping Research on Track’* describes six main values to guide research with Indigenous people: Responsibility; Respect; Survival and Protection; Reciprocity; Equality; and the overarching value that projects be conducted in the right Spirit and with Integrity [10]. It describes eight steps in the research process from conceptualisation to dissemination of the results and suggests a number of important questions for communities to raise during the research process such as ‘Are the researchers respecting our values and ways of doing things?’; ‘Are there appropriate community/organisational consent processes in place?’; and ‘Is this research a priority for our community/organisations?’ [10]. Although other documents describe the values needed for ethical research with Indigenous communities, no publicly available documents describe in detail how to seek consent and ensure that the research information is understood [3]. The Lowitja Institute’s guide: ‘*Researching Aboriginal Health: A Practical Guide for Researchers’* [4] describes issues that researchers need to be aware of when working with Aboriginal communities such as ‘sorry business’ a special mourning period where the family of the deceased are not to be disturbed; and ‘men’s business’—private discussions amongst males on issues such as men’s health or cultural lore, where women are forbidden to be involved. The Lowitja Institute gives examples of locally designed projects that use a variety of different media to communicate information when seeking consent [14, 15]*.* However, the effectiveness of these approaches is not evaluated [4, 14, 15].

The Picture Talk Project included a systematic review [3], interviews with Aboriginal community leaders (reported in this manuscript) and focus groups with community members. The Picture Talk Project follows the recently completed Lililwan Project, a study of Fetal Alcohol Spectrum Disorder Prevalence that was initiated by and conducted in partnership with Aboriginal leaders [16–19]. In our recent systematic review, we identified few studies that evaluate the community engagement or consent process with Aboriginal communities [3]. The current paper reports upon findings from interviews with leaders of Aboriginal communities which aimed to explore the experiences, attitudes, and understanding of Aboriginal people of the Fitzroy Valley of research, the process of community engagement, consultation and obtaining consent, and the individual consent process for research.

## Methods

This research is reported in line with the 32-point checklist in the consolidated criteria for reporting qualitative research (COREQ) guidelines [20].

### Population

The Fitzroy Valley is based in The Kimberley, northern Western Australia with a population of approximately 4 500 people, 80% of whom identify as Aboriginal [21]. The town of Fitzroy Crossing and the 45 communities surrounding it in a 200 km radius are all classified as ‘very remote’ [21]. There are four dominant Aboriginal language groups within Fitzroy Valley communities, these are Bunuba, Walmajarri, Wangkatjungka and Gooniyandi. Other language groups include Kija and Nyikinya. These groups have distinct cultural and linguistic characteristics and for many, their first language is their Aboriginal language. Other languages spoken include Kimberley Kriol, Aboriginal English and Standard Australian English.

### Invitation

In 2011 Aboriginal leaders of the Fitzroy Valley invited a project to reflect on the process of community engagement, consultation and consent for research, which they called The Picture Talk Project. This was named a local Aboriginal leader Marmingee Hand in reference to the pictorial flip card that was used in conjunction with the standard consent form by a local interpreter when seeking consent for The Lililwan Project [18].

### Researcher background

It is important to consider the lens through which qualitative research is conducted [20]. The lead author EF is of Irish heritage, was born in England, is the oldest of six children and moved to Australia when she was a child. EF completed all of her medical training in Australia (BMedSci(Hons), MBBS, DipCH) and is specialising in paediatrics. This research is part of a PhD at the University of Sydney. EF joined a qualitative research support group and attended qualitative research training courses prior to commencing this project. She is supervised by researchers who are experienced in qualitative research, paediatrics, psychology, public health, anthropology and cultural protocols of Aboriginal communities. EF has some bias in favour of the Lililwan Project as she was directly involved and witnessed the positive research relationships with the community [16–19]. As a doctor and a researcher she is passionate about achieving health equity for all Australians.

### Cultural capacity training and research partnerships formed

EF and other non-Aboriginal members of the research team received cultural awareness training from Nindilingarri Cultural Health Services. EF and EE worked with JO and MC and other Aboriginal community members over several years as members of the Lililwan Project research team [16–19]. Through this process they formed strong trusting relationships with the Aboriginal community members and gained respect from community leaders for The Picture Talk Project. A partnership was formed between Aboriginal leaders in the Fitzroy Valley (JO, MC and TL) and researchers in Sydney and Darwin (EF, EE, AM, and HD’A a senior Aboriginal researcher).

### Community engagement, consultation and consent

Aboriginal community leaders invited EF to talk about The Picture Talk at local meetings such as The Fitzroy Valley Future’s Forum [22]. In this way, The Picture Talk Project was introduced to the wider community. Information was presented by EF in plain English using a power point presentation containing text and graphics in partnership with a local Aboriginal research team member.

**Fig. 1:**
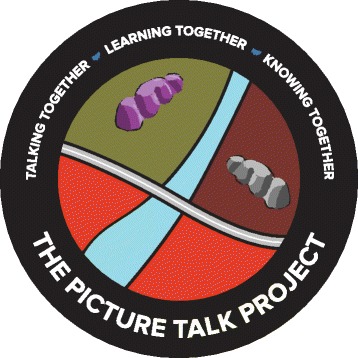
The Picture Talk Project Logo. In the centre of the logo, the historic Fitzroy Crossing is shown at the crossing of the river (*blue*) and the highway—symbolising the meeting of the Aboriginal world with the Western world. The quadrants this creates represents the main language groups and regions of The Valley—the purple hills of the Leopold Ranges in the north for Bunuba country; the *Black Hills* in the east for Gooniyandi country and the Great Sandy Desert in *red* in the south for Walmajarri, Wangkatjungka, Nyikinya and Kija country. The *black band* surrounding the land conveys the message that in order to work with the Aboriginal communities of the Fitzroy Valley, one needs to enter through the local Aboriginal organisations and work in partnership with local Aboriginal people

A project logo (Fig. 1) was designed with Community Navigator Sandra Nuggett and local Aboriginal artist Neil Carter at the Kimberley Aboriginal Law and Culture Centre (KALACC) in order to make the research team recognisable and capture the spirit of the project. The motto on the logo is ‘Talking Together, Learning Together, Knowing Together’.

### Community navigators

Respected Aboriginal researchers with local language and cultural knowledge were recommended by community leaders and employed as Community Navigators to guide non-Aboriginal researchers through the cultural protocols of engaging and working with the community. During interviews with Aboriginal community leaders, different Community Navigators were employed to communicate with community leaders from each language group and male and female participants. Community Navigators worked with EF to recruit research participants and taught EF about local cultural protocols, at the same time gaining skills about Western research techniques. Through this process a two-way community-based participatory research partnership was formed [23].

### Individual consent

Consent was obtained from all Aboriginal community leaders who participated in this study. The study details and consent procedure were explained using a Participant Information Statement (which they could keep) and an Interview Consent Form typed in plain English. A Community Navigator (who the Aboriginal community leader indicated they were comfortable to work with) interpreted these documents into the language preferred by the leader if required. Consent was sought for participation in the interview and for responses to questions to be recorded and transcribed. Consent could be given in writing or verbally, which was witnessed. The option of refusing to be recorded or to withdraw at any time was made clear. Avoidance of the research team was considered refusal of consent from participants. This method of refusal is common among other Indigenous communities [14]. Confidentiality about participants and secure storage of data was assured as part of the consent process. Contact details were exchanged to allow for questions and feedback of results. A short, structured questionnaire was used to obtain the participant’s age; language group; preferred language; educational level; and role in the community, e.g. CEO of a community organisation or a cultural role such as being responsible for taking men through cultural lore. Leaders were asked if they had been involved in the Lililwan Project consent process. Community Navigators could be approached any time to answer questions or accept feedback that Aboriginal community leaders wanted to give indirectly to researchers,—a locally respected process known as ‘talking sideways’. Given the importance of these participants as leaders of the Fitzroy Valley community, we refer to participants as ‘Aboriginal community leaders’ for the rest of this paper.

### Recruitment to participate

Recruitment was conducted through stratified purposive sampling and snowball sampling [24–26]. The stratified purposive sampling methodology was utilised and guided by Community Navigators [24–26]. It was considered important to have both male and female participants, elders and Aboriginal community leaders from each of the four main language groups of the Fitzroy Valley. Snowball sampling occurred when friends and relatives of the Community Navigators and local Aboriginal Chief Investigators were invited to participate when they approached the research team in person to ask about the project [24–26]. The research team ensured they were available for further questions and remained flexible with time, so potential participating leaders could consult whoever they needed to and consider if they wished to participate. Aboriginal community leaders were not approached if they had cultural obligations such as ‘sorry business’ (mourning), as advised by the Community Navigators.

### Data collection

Semi-structured interviews about community engagement and consent for research were conducted with Aboriginal community leaders in the style of ‘research topic yarning’. This involves a semi-structured interview presented in the form of yarning which is described by Aboriginal researchers such as Bessarab [27] and Geia [28] to be similar to a conversational format that is relaxed and reflexive to what the respondent says. The interview is not bound by a strict series of ordered questions but is guided by topics of conversation. This allows the interview to flow more naturally. It gives interviewees time to answer questions through story format if they wish, which is in line with the traditional way of sharing knowledge in Aboriginal culture. Research topic yarning is considered to be a culturally appropriate methodology for working with people who identify as Aboriginal [27, 28]. Interview questions (Additional file 1.) were based on the literature on qualitative research [23–25, 27–31]. Questions were informed by a systematic literature review [3] of international, national and local research publications and guidelines on conducting research with Indigenous communities [4, 8–13, 32, 33]. Questions were edited and approved by all chief investigators and JO and MC ensured the language used was culturally appropriate and easy to understand for people for whom English is not the first language. The interview was pilot tested and refined. Each question was read out loud by EF and interpreted into the Aboriginal community leader’s language of preference by a Community Navigator if requested. Questions were predetermined, however interviews were kept flexible to allow for a natural flow. Time was allowed for silent pauses after questions to permit participants to collect their thoughts and respond when they felt comfortable. All responses were encouraged even if they did not seem to answer the question at the time. Interview responses were voice recorded and transcribed afterwards or documented at the time if a participant declined consent to be recorded. If consent was granted, the voice recorder was turned on in front of the participant to indicate the start of the interview and placed under a piece of paper to help participants relax when answering questions without the visible presence of the recorder [34]. Participants could nominate the place and the time for their interview. The aim was to collect data until all themes were ‘saturated’ [29].

Community leaders were interviewed about:

- Their understanding of and past experiences with research
- Advice for external researchers on the best way to seek consent for research
- Who researchers should work with when conducting research
- The role of community elders in research projects
- Appropriate ways of disseminating research information for:
  - ○ Seeking community consultation
  - ○ Seeking community consent
  - ○ Recruiting participants to a study
  - ○ Reporting results back to the community
- The process of confirming consent for research
- General advice for future research

### Data analysis

Interviews were transcribed into Microsoft Word and transcriptions were then checked against original audio files for accuracy. Transcripts were made available for comment or correction by participants. Transcripts were uploaded into NVivo10 Qualitative Software that was used to facilitate coding [35, 36]. Initial thoughts about the main points raised during the interviews were documented by EF in a research diary immediately following the interviews and during the transcription and crosschecking process to allow consideration of interview responses in their original context [25, 26, 29, 36, 37].

A topic guide was formed for the initial stages of analysis of interview transcripts using NVivo10 Qualitative Software [35, 36, 38]. Data were coded using an integrated approach, combining deductive and inductive methods [37]. References to a specific theme, person, place or other topic of interest were collected into basic units of data called ‘nodes’. Nodes that were titled ‘research’ and ‘consent’ were deduced from interview questions in order to further explore community leaders’ understanding of and attitudes to these topics. Remaining nodes were created inductively using grounded theory in order to enhance creativity and find deeper meaning in what Aboriginal community leaders were saying [37–39]. Some nodes were created based on first impressions; others during crosschecking of interview transcripts; but the majority of nodes were derived directly from coding the interview transcripts in an iterative process [24–26, 29, 36–39].

Interview transcripts were coded line by line into nodes. Preliminary nodes were structured into a hierarchy of ‘parent’ and ‘child’ nodes and formatted as more interviews were coded. Parent nodes represent general topics, encompassing child nodes of more specific subcategories. Nodes that were very similar were combined and nodes containing grouped data that could be separated into different topics were divided. The node hierarchy was then finalised [24–26, 29, 36–39] and to ensure that all text was coded into the finalised nodes, all transcripts were then re-read and re-coded [24, 37–39].

Throughout the process of coding, themes emerged from the data. Themes varied from topics that were discussed by many of the interviewees to topics that were highlighted as very important to the community. Themes were compared constantly and recursively back against the data—a process described as the ‘constant comparison method’ to ensure they were not taken out of context [25, 38]. Themes were then further analysed in light of the research questions for this study and to generate new interpretive conclusions [24–26, 29, 36–39]. Rich quotes were extracted to exemplify themes. The data collection and coding process was considered complete when themes were saturated and no new topics arose from the data [24–26, 29, 36–39].

For consistency, EF coded all transcripts. To ensure reliability and cultural safety with coding and minimise misinterpretation, sample extracts were coded by a Community Navigator and verified against EF’s coding [37]. To ensure findings were communicated in a culturally appropriate way, results from the analysis were checked by all Aboriginal researchers who are chief investigators of this project. Research participants also provided feedback on the findings.

**Fig. 2:**
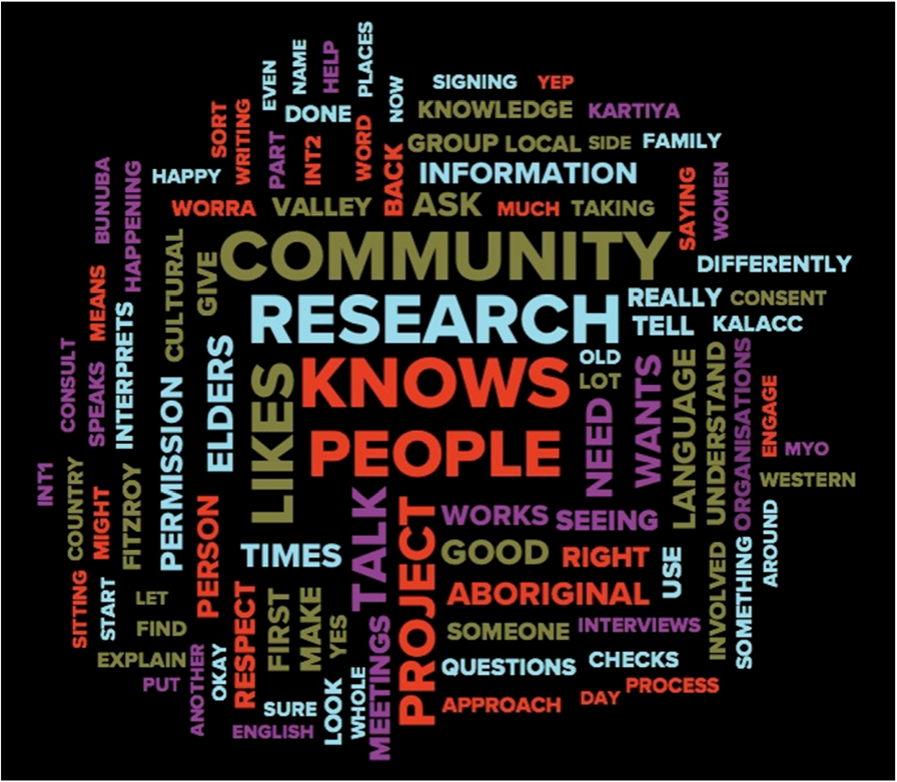
Word frequency cloud created using NVivo10 qualitative software

In addition, a ‘word frequency cloud’ was produced using NVivo10 Qualitative Software (Fig. 2). All interview transcripts were scanned for all words and their derivatives greater than two letters to see which were used most often. This produces a word frequency cloud to visually represent results. The larger words are words that were mentioned most frequently by Aboriginal community leaders.

### Ethics approval

Consistent with NHMRC guidelines for conducting research with Aboriginal communities, the Picture Talk Project upholds the six core values listed as important, namely: Respect, Equality, Reciprocity, Survival and Protection, Responsibility and above all that the project be conducted in the right Spirit and with Integrity [9, 10]. The project is supported and guided by leaders of the Aboriginal communities of the Kimberley; three of the Chief Investigators are Aboriginal women and one co-author is an Aboriginal man who leads the Kimberley Aboriginal Law and Culture Centre. Ethics approval was granted by the University of Sydney Human Research Ethics Committee (No. 2012/348, reference:14760), the Western Australian Aboriginal Health Ethics Committee, the Western Australian Country Health Service Research Ethics Committee (No. 2012:15), and the Kimberley Aboriginal Health Planning Forum Research Subcommittee (No. 2012–008).

## Results

The strategic purposive and snowball recruitment of participating Aboriginal community leaders through Community Navigators was effective in the Fitzroy Valley due to the strongly connected community network. There was a 95% consent rate from Aboriginal community leaders invited into the study. Twenty leaders of the Fitzroy Valley were interviewed and their demographics are shown in Table 1. One leader declined consent explaining that he did not have time to participate in the interview. Leaders came from all (Bunuba, Walmajarri, Wangkatjungka, Gooniyandi and Kija) language groups and only 30% preferred to talk in Standard Australian English. Equal numbers of men and women participated, including a number of elders (*n* = 6). Participants played multiple roles within the community, including: cultural advisor; chairperson or chief executive officer (CEO) of an Aboriginal community-owned organisation (specific organisations are not identified for confidentiality); artist; interpreter; and custodian of knowledge about bush food or traditional medicine. Education level attained varied from primary school completion to university qualifications. Interviews ranged from 20 to 140 min in duration with an average of 54 min.

**Table 1:** Demographics of Aboriginal community leaders participating in interviews (*n* = 20)

Participant Demographics		No.	%
Sex	Male	10	50
Identity	Aboriginal	20	100
Age	31–40	2	10
	41–50	5	25
	51–60	9	45
	61+	4	20
Language Group^a^	Walmajarri	9	45
	Wangkatjungka	2	10
	Gooniyandi	4	20
	Bunuba	6	30
	Kija	1	5
	Other	2	10
Preferred Language^a^	Standard Australian English	6	30
	Aboriginal English	1	5
	Kriol	2	10
	Walmajarri	10	50
	Gooniyandi	1	5
	Bunuba	3	15
Cultural Knowledge^b^	Elder	6	30
	Cultural Advisor	5	25
	Chair Person	7	35
	CEO	2	10
	Legal Advice/ Governance structure	2	10
	Interpreter	3	15
	Guide for Non-Aboriginal people	6	30
	Bush-food/ Hunting/Fishing	4	20
	Teaching the children	5	25
	Grandparent/ Parent	7	35
	Art/ music	3	15
	Health	1	5
	Sport	1	5
Education	Did not say	3	15
	Primary	4	20
	High school	6	30
	Training Courses	3	15
	University	4	20

^a^Some Aboriginal community leaders identified with two or more language groups and preferred different languages depending on who they were talking to

^b^Cultural knowledge was also considered important to the community

### Interview setting chosen by Aboriginal community leaders

Aboriginal community leaders were given the choice of where and when they wished to participate in an interview. Interviews were conducted in a number of settings including: at a local community organisation; in the privacy of an office or in the front yard of a person’s house and sometimes witnessed from a distance by family or local community members. One interview was conducted with two leaders together as was their preference. Witnessing is an important part of research in oral traditions and provides a balance between confidentiality and having someone vouch for the fact that the research is legitimate. Community leaders explained the need for a witness present during an interview:

*So when talking with Aboriginal people we also have to be mindful of the people present at these places where we might be engaging in conversation, the types of questions that are asked, who’s there? It’s important to have witnesses there to verify what it is you’re being asked and how it is you’re responding to those questions…They may want to have the witness that I talked about, someone there to verify that the researcher has engaged with the person in a respectful way and the local person has participated and willingly involved themselves in the research.* (Participant 18)

Having a witness present during research proceedings provides secondary evidence for the manner in which research was conducted based on the witness’ perspective. It may affect the results as participants may share different information in an interview depending on who is present. Concerns about confidentiality were raised by EF when a participant requested that there be a witness present for the research proceedings, usually a family member with experience working in the Western world. They did not directly participate but were within earshot of the conversation. Older people who are not strong in English have a strong preference for someone else to be present to interpret as well as witness proceedings so that they are not deceived into consenting to something that is against their wishes. This request was honoured in respect for these conventional cultural protocols.

Grounded theory was applied to the research process enabling findings or preferences from initial interviews to be fed into the project design [24, 26, 30, 37, 38]. This helped ensure that proceedings were culturally respectful.

### Themes

Five themes were extracted from the interviews, namely: Research—finding knowledge; Being respectful of Aboriginal people, Working on country, and Being flexible with time; Working together with good communication; Reciprocity—two-way learning, the community must benefit from the research; Reaching Consent—who gives permission and how? These will be elaborated upon below. Quotes (in italics) from participating Aboriginal community leaders were extracted from interview transcripts to support these themes.

1. Research—finding knowledge
  What is Research?
    Participants were asked if they had a word for ‘research’. Some explained there is no word for research in their language and that they had very limited exposure to Western research:
*I never grew up knowing the word research until I got into adulthood… research is a very Western thing about learning about new ways of working or getting new information on what it is that affects people.* (Participant (P) 20)
    One Aboriginal community leader was able to identify a similar word in a local language:
*What is research, like looking on things, looking, checking? In Walmajarri I would probably say ngalaramarnu – Looking for more .. and parlipungu – finding out about things.* (P16)
    Others described research as:
*It’s finding out information and it’s not just about asking questions, it’s having conversations and getting feedback and getting people’s ideas.* (P18)
    Another Aboriginal community leader linked knowledge from research with oral tradition and connection with country:
*Well it’s just sharing of knowledge. We share by word of mouth and I guess the Western culture has it in writing to remind them but we have seasons for that.* (P19)
  Positive Experiences with Research
    Many Aboriginal community leaders had been involved in research projects. Positive experiences were described when researchers had consulted the community about the research; worked with local Aboriginal community members and paid them for their time; learned about the cultural diversity of the communities of Fitzroy Valley; had good communication; and developed outcomes which benefited the community. One example was in regard to working with an anthropologist:
*The anthropologist that we had knew and understood the way our kinship people worked and he understood the culture diversity among our own people and he knew the stories, the live stories and he knew about each of the sacred water holes and the names to them.* (P9)
    Another example was The Lililwan Project on Fetal Alcohol Spectrum Disorder prevalence. At the time of these interviews, the project was nearing completion and was fresh in the minds of many of the community leaders. There were many positive comments:
*The Lililwan thing was done beautifully. I mean it couldn’t go wrong because it did all the right things in the first place… Yeah, it’s a good example of how it can be done and how it should be done and how it was done properly. I’ve never heard anybody complain about the way that whole program worked because it’s valuable information.* (P14)
    It set an example for future research:
*The Lililwan Project… was so successful, because there was a lot of groundwork that took place first.* (P20)
  Negative Experiences with Research
    Some researchers had failed to conduct projects in a respectful manner and were regarded unfavourably by community leaders:
*I did not find them respectful. I had to straighten them out. I found them respectful to decision making but I didn’t like the way they handled/spoke to people. After that we had to keep reminding them. You can’t just come into an Aboriginal Community and start talking to them. You have to let them know who you are, what you’re doing, what you’re talking about. Send a fax or email. Most communities have a phone, fax and email.* (P7)
    Past studies have failed to involve community members in the research process:
*But in the early years I’ve known that people have done research and didn’t really involve the community in a better way. You know like not explaining what the research is about and they just did it anyway because it’s a government… it should be working with the people, you know because people have the knowledge.* (P16)
  Why do research?
    There was a general consensus that research was important for the community.
*It’s important for people… to understand what the research is about and how it’s going to benefit them. And more importantly too I think is the ownership of the material too, that’s pretty important. Because some of that information that’s gathered from research, data and all that is beneficial to community groups and organisations, they have to be able to use too for their own needs, you know. Whether it’s lobbying governments for better services … information is important.* (P14)
    Research can be very taxing on a community’s time and resources, it is important that it is meaningful to the community:
*It’s one thing to do research, okay how do we use the research then to deliver to these others, leverage in terms of bureaucracy? And to make changes in the policies that delivers the funds to deal with the different issues. So yeah I guess everybody wants research not just for the sake of research, but for change.* (P19)
2. Being respectful of Aboriginal people, Working on country, and Being flexible with time
  Aboriginal community leaders said it was important for researchers to: treat all members of the Aboriginal community with courtesy and respect; avoid talking down to them in academic language; respect the need for people to be on country, in their domain; and respect people’s time. They also said it was important to pay the Community Navigators for their time; give people notice of when they are coming to the community; and to be patient while waiting to hear back from people while they consider consenting to a project.
  Respect Aboriginal communities and their knowledge
    Aboriginal communities have a lot to offer and researchers must respect their expertise:
*So if there’s research to be done that impacts this community, that affects this community, then we need to understand from the outset that the community… is a reservoir of knowledge and there’s many people with varying depth of knowledge you know depending on their age and their experiences and their stories, their histories which all are legitimate and play an important part in the telling of the story of that community or that issue that is of interest to the researcher and to the community members.* (P18)
    In relation to the Lililwan Project one Aboriginal community leader said:
*Yeah, well for me I think that we all came together and we were all respectful of who we are as human beings and that we all came with a set of skills that joined part of the jigsaw for that study and there was the respect and the sharing of information that went on between us…no one was more better than the other one, that everyone was able to participate and talk freely about what it is that they were, things they were worried about anything or whatever, everyone came to the table being respectful.* (P20)
    It is important to respect the organisations that have been established in the community:
*It’s really common sense but with research they tend to just come in and disregard all that cultural knowledge and local information or there’s no respect for having Aboriginal leaders in the Valley that have that authority and that knowledge.* (P17)
*There has to be respect both ways and good explanations on why a research project is of interest and why the research is there…So I think gone are the days of when people were the subject of research and people weren’t being respectfully engaged in the research and their strength and knowledge wasn’t being acknowledged properly…I think it’s respecting the community, showing … that you’re here to be guided by the community…Communities are tired of researchers coming in and out and having no accountability back to the community… With… relationships I think, the researchers are able to build respect for themselves as researchers and the project and people here locally are able to provide some really honest feedback around that.* (P18)
  Respect people’s connection to country
    Community leaders talked about how important working on ‘country’ is to them and how it is important that there is opportunity to conduct research on the land with which they have ancestral ties:
*I know that Granny M talks about you taking them to the river and sitting down … all these old people they enjoy that because they see that…you’re acknowledging a different way of extracting the knowledge or extracting the information and you’re acknowledging that this is the way the information is going to be given to you and it’s about being on country, their country, and when you’re in that space with them they become your teachers…So it’s around acknowledging that to get information you can’t just be in an office space.* (P20)
  Respect people’s time and cultural protocols
    Leaders say researchers must respect the Aboriginal community’s time, give them notice when they are coming and space when they need it and be patient for a response. They will tell you when they are ready:
*Sometimes they need a letter in advance to say we’ll be visiting your community on so and so day and month and time. That’s what some communities want in place. Before anything happens. So they have time to think about it.* (P2)
*They need to be given time you know to, to make a decision because Aboriginal people are different you know.* (P17)
    Researchers need to understand and respect cultural protocols such as ‘sorry business’ (mourning after the death of a family member). This concept is not unique to the Aboriginal communities of the Kimberley. It is important to respect the protocols around death—not to mention the deceased by name or show images of them and not to ask anything of family members of the deceased until they decide they are ready:
*Sometimes it takes time, some things can slow you down – like sorry business.* (P16)
    Researchers must invest time getting to know the community in order to earn a respectful and trusting relationship:
*Well I think, you know other than getting to understand the history of the area… taking the time to go and engage with organizations and groups for a decent period of time before introducing the research project, because no doubt all of that fact finding, informing of oneself will help shape the topic of the research and so investing in that time is absolutely critical for any successful research project.* (P18)
    Be generous with time—respect the cultural protocols for decision making:
*Don’t keep pushing yourself in, saying it’s all about your work so you can finish your job. Saying ‘I need my job finished I need to come’. If you start pushing yourself in, they’ll never let you come in. Because they know you’ve got your work but what about our side of work, you’ve got to show respect, show respect to their side.* (P8)
    One elder was very passionate in response to questions about the timing of when research should start. We were interpreting the questions and giving supportive information when this was met with silence, then she interrupted mid-question in English:
*When they’re ready!!* (P12)
3. Working together with good communication
  The community leaders of the Fitzroy Valley emphasised the importance of equal partnership in the research process.
  Equal partnership in the research process
    Having local Aboriginal people join either as chief investigators or research team members on a project was described as essential:
*I was really pleased that we were able to participate and really shape that research project* (The Lililwan Project) *so that everyone who should be involved were involved and it was done in culturally appropriate way. I felt it was really successful because it was a very important topic and I wanted to do things differently to how research was done in the past.* (17)
*l don’t think researchers should do research with any Aboriginal people until, unless they’ve engaged fully with the Aboriginal people from the start to the end.* (P17)
*So they should have community supervisors alongside academic supervisors. So that…, people can liaise to discuss the research and their conduct of the research project and provide the guidance needed by the researcher.* (P18)
*Aboriginal people have to always be a part - that they’ve got to be equal partners in the research, not as the person that’s being researched. They’re equal partners in research.* (P20)
  Being introduced to communities
    There are cultural protocols underpinning working with communities. Better relationships are formed with Aboriginal people when someone they know, in this case the Community Navigator, introduces a new person to a community. The Community Navigator should be someone they respect; ideally from their own language group or who has good kin connections; and usually someone of the same sex.
*The local* (Community Navigator) *will explain what you’re doing there what’s your role in this. It’s best to work with locals, or Navigators, once they start to introduce you to all those communities then you can work on your own, because they know who you are.* (P8)
  Starting the conversation
    Leaders give advice about how to approach communication with an elder for research:
*Some Aboriginal person who knows which elders that you need to go and sit down and engage and have a conversation and have a cup of tea … You get more out of them than… having a formal interview, discussion. So you first go informally, sit down you know. Make contact.* (P9)
  Explaining the research so that people understand, check that they do
    Leaders say it is important to present information about research in simple terms, using words or pictures and an interpreter. Repeat presentations multiple times. Check that people understand, either by asking them or through the Community Navigator. Be open and available to answer any questions.
*Some understand, sometimes you have to go back again and talk to those people. In 1 day it’s really hard, to go and sit with that person because that person might have something on his mind or not really clear about it. You have to go back again and sit down and talk to those people, understand?* (P8)
    Indirect feedback is called’talking sideways’, and Community Navigators have an important role in collecting this feedback e.g. when seeking consent:
*Yeah it’s talking sideways. That’s why we go back and explain to that person what he doesn’t understand. So that person might come up and say ‘I don’t understand this and that’, so you explain it to them again. Make sure they fully understand. Before asking them to sign.* (P8)
    Often organisations come to the community with presentations but fail to answer or follow up on questions:
*We call them ‘fork-tongue’ – come here with one word and go there with another word. That’s what’s been happening. A lot of people got confused. So when they wanted us to decide* (to participate In research)*, people didn’t want to decide because some people really didn’t understand what they were saying, so we call them fork-tongue. You’ve got to be open, you know, we really understand when you’re open. Don’t say ‘ah we’re going to have a meeting with you’ and talk about this and that and then come the questions they say ‘Ah I’ll have to get back to you on that’. I’d rather they have the answer. That’s not really good.* (P8)
  Be aware of your body language
    Community leaders gave advice as to how to communicate with elders:
*It is important to work with Aboriginal people and have respect. Come down to their level – sit on ground. It is good that you have an interpreter with you.* (P4)
    Aboriginal people often avert their gaze during conversations. One leader specified the context in which it is OK to make eye contact with Aboriginal people, namely while conducting *‘Kartiya business’* meaning Western research. There is an awareness of the Western approach to communication and this is accommodated for by Aboriginal people.
*Are you okay with my body language now? Is my level of eye contact appropriate?* (EF)
*Yeah, we doing Kartyia business.* (P10)
    The way Aboriginal community members talk together is to limit eye contact with senior people.
*I know in the West they (make eye contact), and I’ve had it when I was in school. If you’re not looking at the teacher, he automatically assumes that you’re not listening. But that’s not necessarily the case. Your ears are on the side of your head…I know with our people here in the Valley, I know a lot of traditional people, you don’t necessarily have to look at them. That Western onus of looking a person in the eye if you’re speaking to them doesn’t necessarily apply… with traditional people. It’s actually rude to be looking at them. You look down, you just talk. You can glance at them every now and then… It’s a bit of a clash, there are two ways of communicating. In a Western way there’s, ‘you look at me when I’m speaking to you’, you know.* (P14)
    Dressing conservatively is essential to showing respect to the community:
*Sometimes we have people come up here, all with good heart … from Perth or somewhere and they just come out of that metro lifestyle and they think they can walk in here with stockings and short skirts and we all just sit there and go: can somebody tell this person, please this is not appropriate for up here.* (P11)
4. Reciprocity—two-way learning, how does the community benefit from the research?
  Community leaders of the Fitzroy Valley feel strongly that all research done in the Valley should be meaningful to the community; the community should have ownership over the project; and results should be used to benefit the community. Aboriginal organisations and Community Navigators put a lot of energy into training researchers to respect cultural protocols. At the same time researchers should provide direct benefits to local people through employment, training and experience, and acknowledging them through payment and authorship in publications.
*I think at the end when the community welcomes you back to give feedback and that’s done in a respectful way, I think that’s an indication that you’ve done right thing with the community.* (P17)
  The community is motivated to take part in research that addresses important issues within the community. An Aboriginal community leader talked about how the community problem of fetal alcohol spectrum disorder was first raised at the traditional Women’s Bush Meeting, which is held out on country:
*I believe that if you’re going to do research … we should benefit you know. There’s only one research project that I think we’ve benefited from and that’s the Lililwan project.* (P17)
  Cultural capacity building—for the Non-Aboriginal researchers
    In order to understand cultural protocol it was recommended that researchers have training:
*Go through any cultural training program, that’s a must, they need to have that because it’s a learning curve for everybody. Us as Aboriginal people and the researchers that are coming in as well.* (P9)
  Research capacity building for Aboriginal communities
    In the same way that Aboriginal people are cultural guides for Non Aboriginal researchers, it was important that Western research approaches and knowledge were shared through the process:
*Well first and foremost, preferably engage with an organisation, like you have, and then secondly use youngsters in the process. Not only to transfer your Westernised skills but to also utilise their skills in dealing with our members as well.* (P19)
5. Reaching Consent—Who has the voice? Who gives permission and how?
  Informed consent is founded on the principle of autonomy, the right of an individual to make decisions about his or her own person [3]. This Western doctrine stands in stark contrast to Australian Aboriginal values, where the family unit and community needs are at the core of the decision making process [3, 4]. When it comes to giving permission for research there are a number of players involved. Aboriginal community leaders each have different knowledge, power and responsibilities. Elders are the important people in the Aboriginal cultural hierarchy. They hold the knowledge of country and the people; they are the keepers of cultural protocol. People in the ‘Karrayili’ age group are ‘middle aged people’ on their way to being elders and they also hold many community responsibilities. Other leaders include Chief Executive Officers of Aboriginal Non-Government Organisations or chairpersons for Aboriginal communities. They hold knowledge of Western protocol and work in both the Western and Aboriginal worlds. In addition, Community Navigators were employed by The Picture Talk Project to broker relationships between Non-Aboriginal researchers and Aboriginal community leaders. They are people who hold local respect, Western knowledge and Cultural knowledge and could interpret English into a local language. The community leaders of Fitzroy Crossing explain the importance of these roles below. They emphasise that community consent and consultation must be sought in addition to individual consent. People won’t sign unless they know who you are, what are your intentions and are you trustworthy. By forming respectful relationships; designing the research together; giving people time to think about participating; working on country; and having good open communication; Aboriginal people are often willing to be involved. The process of seeking consent is continuous throughout a project.
*They should first have the discussion with the community about their idea…, of wanting to do research. So the community should be informed from the beginning… and be involved in how it should be done and be guided by their Aboriginal community to. do so If it’s research about the whole Valley then it’s important to speak to the four language groups or certain significant people within those language groups who will then say go and talk to this community and go and talk to thing and then from there you’ll be given permission to talk to others.* (P17)
*Ask the chairperson who will then present it at a meeting and discuss it with the community members. Once they say yes then you can talk to the community members.* (P4)
*So yeah, community has to have a big say in the research and if the community says no well then so be it.* (P20)
  Elders must be consulted for consent for research with the community
    Talking about the role of elders with research projects:
*They’re the keepers of knowledge and they pass that information down and they continue to be our teachers and keep us on the right track when it comes to how we live and how we conduct ourselves around here because like people say we live in two worlds so we got to try and find a balance.* (P20)
*I think it’s important to acknowledge that elders have that cultural authority and that they can make decisions for the whole of the Fitzroy Valley.* (P17)
*Elders, they are the driving force behind the community. They have more knowledge than anyone. They are the keepers. They hold everything together. All ladies all men, they need to be involved.* (P7)
*They have the knowledge of bush, animals and plants.* (P4)
  There are other cultural leaders that are ‘Middle of the road’ on their way to being elders
    These people are also important to consult when wanting to engage with elders and the community for research:
*Well yeah, I mean well I’m getting to that Karrayili age now that people are starting to see me as that, you know. But I don’t see myself as an elder because I always refer back to my older thing you know… that middle of the road, which is Karrayili.* (P9)
  Community Navigators need to be employed to assist in seeking consent
    When working with elders, researchers are advised to work with an interpreter or Community Navigator. This allows questions about the research to be asked at a later time, in an indirect approach through people that the community know well and trust:
*Better with an interpreter to make sure they understand… It is very good to come with a Community Navigator. It is the same with all the old people. She (the Navigator) can answer questions they may have at the time or after.* (P4)
*English is probably their third or fourth language, and sometimes they might not understand English. But it would be good to get someone to sit with you talking to that person.* (P15)
    The role of the Community Navigator goes beyond just interpreting—there is a formal introduction:
*He’s like a key, he is opening the door for you. He helps connect you in the right way. He helps explain why you are here and what you want to do.* (P7)
*I think you should be guided by the Aboriginal person that you’re working with, they’ll know you have to be careful. It depends on what the topic is and the cultural protocols around and how you engage with men and how you engage with women. So you know if you got, if you’re working alongside an Aboriginal person they would be able to navigate that for you and assist you with that.* (P17)
  Local Aboriginal Non-Government Organisations are the first port of call
    Elders and chairpersons of communities are connected through local Non-Government Organisations:
*Well the first thing is you come* …(to KALACC)*. And depending on what job you are doing for what language group. See the elder from there or chairperson.* (P5)
*Yeah a lot of the old people, a lot of the elders and a lot of the leaders in the Valley are involved in more than just one organisation. They’ve all got their feet and legs and hands in camps of other business too.* (P14)
  When reaching the point of giving consent, Aboriginal leaders want it in writing:
    For initiating community consent or when seeking individual consent, most of the Aboriginal leaders preferred to have a witness present and give signed consent over verbal consent:
*I prefer written…some people don’t know how to write their name.* (P8)
*Because it’s oral, you need someone to witness them, so the next time you come around they might say no I have never seen those people and you’ve got the witness there.* (P8)
*I encourage people to you know, get things in black and white as evidence… individuals maybe different but from my side I certainly push for written consent. That way it protects the individual if anything comes out.* (P15)
*Yeah, I’d rather sign… Because if you say ‘oh yeah, I agree’ and you had it on a recorder, it could be anyone’s voice really.* (P20)
  Direct feedback for The Picture Talk Project methodology
    Aboriginal community leaders gave direct positive feedback. They used the way in which The Picture Talk Project research team were conducting the interview as a practical example of culturally respectful research:
*It is good that you have an interpreter with you. Interpreters are really important to the Valley. (P4)*
*Like what you are doing. Getting to know PB first. Coming to KALACC first. Getting to know the Aboriginal organisations. Getting the feel of it. You’re staying in the community for a while. The environment of the local people. It makes you more confident going out.* (P7)
*I’ve seen you in and out of the office, which makes me more comfortable to speak.* (P7)
  Word Frequency Cloud
    A word frequency cloud was created using the NVivo10 Qualitative Software (Fig. 2). The words that were mentioned the most frequently by Aboriginal community leaders were ‘community’, ‘research’, ‘knows’ and ‘people’.

## Discussion

As indicated by our systematic review, few studies evaluate the consent process for research with Indigenous communities [3]. The Picture Talk Project is the first study of its kind, specifically addressing Aboriginal leaders’ knowledge and attitudes to community engagement for research and the consent process.

The key findings from interviews of 20 Aboriginal leaders during The Picture Talk Project are exemplified through the 5 major themes:

1. *Research – finding knowledge*: There many experiences and understandings of what research is and why it is conducted. The word research does not exist in some Aboriginal languages
2. *Being respectful of Aboriginal people, Working on country, and Being flexible with time*: It is imperative that researchers work respectfully with Aboriginal communities, and recognise the value of working on country, being flexible with time and acknowledging cultural protocol;
3. *Working together with good communication*: By designing a project together with Aboriginal communities, it will be conducted in a culturally sensitive way;
4. *Reciprocity – two-way learning*: It is important to provide research skills for local Aboriginal people involved with the research and cultural education for the Non Aboriginal researchers including how to approach, and communicate more effectively with Aboriginal community members. It is also crucial to report research results back to the community, so that their contribution can be acknowledged and that research findings benefit the community.
5. *Reaching consent*: Only when all of the above are adhered to it is possible to obtain informed consent from the community and individual participants.

As shown in the word frequency cloud in Fig. 2 the Aboriginal community leaders of the Fitzroy Valley place a large emphasis on the *community’s role in research* and brokering this through relationships with *people such as elders and other community leaders who know* about culture and country.

One of the main messages from community leaders was that researchers will not gain consent for research unless the community is fully engaged from the start to the completion of a project. Ideally, the research question comes from and is prioritised by the Aboriginal community itself, as exemplified by the Lililwan Project [16–19].

In order for Aboriginal communities to feel empowered to initiate research, a solid understanding of research is required. Aboriginal community leaders gave a variety of responses about their understanding and experiences with research, and some reported the word did not exist in their language. Similarly, James et al. reports that when Native American participants were asked about research they were frustrated because they believed the concept of research was embedded solely in Western values [40]. Some Aboriginal community leaders in the Fitzroy Valley denied any research experience, but went on to describe various science, art and land-rights projects with which they were involved. One leader described her understanding of research as finding ‘your’ knowledge, not necessarily ‘new’ knowledge, implying that there is inherent knowledge that warrants detailed exploration and that may provide valuable insights into the problem in question. Another Aboriginal community leader supports the idea that the knowledge was ‘always there’, it’s just passed down through the generations. Another way this might be interpreted is that through self-reflection, one’s biases and assumptions could be identified and addressed and then the answers to problems may become clear. This is also noted in other Indigenous research paradigms. From his book ‘Research is Ceremony’, Wilson notes that: *‘Research is all about unanswered questions, but it also reveals our unquestioned answers’* [41]*.*

Western researchers often fail to consider the possibility of equally valid research methodologies developed by other cultures. With such assumptions, one may miss the opportunity for research approaches that are novel and potentially more successful. When interview questions were asked in this study, a story was often given in reply. At the outset we often assumed that the Aboriginal community leader had not understood what was being asked. However, with patience and remaining silent it became clear that through these stories, one could identify answers to the questions being asked. In fact, the story could be considered a richer response, as it was surrounded by context, culture and community links. This type of response is quite common among other Indigenous peoples. Tafoya said:*‘Stories go in circles. They don’t go in straight lines. It helps if you listen in circles because there are stories inside and between stories and finding your way through them is as easy and as hard as finding your way home. Part of finding is getting lost, and when you are lost you start to open up and listen’* [42]. The way of answering questions is different among storyteller cultures.

Research projects that worked well in the community had common features e.g. researchers took time to understand the culture and engage the community before conducting research; the most well respected studies employed local people, providing employment and research capacity building [3, 4, 14–19, 23, 27, 28, 40–46]. Participants mentioned they were pleased with the way they were engaged for The Picture Talk Project and said that it was being done ‘the right way’. Bull quotes a Canadian Aboriginal participant who echoes this value of reciprocity: ‘The gain or benefit to the community does not have to be a direct one from the actual research: “Maybe they should do volunteer work in the community while they’re there, or involve students and train them.”’ [43].

Negative experiences with research were often discussed indirectly, without mentioning names. Similar experiences have been reported from Indigenous communities around the world [3, 4, 6, 7, 47–57]. Most projects that did not work well were described as ‘in the old days’, but there were reports of current government-based organisations that were seen to communicate poorly with the community. This may reflect the lack of flexibility in approach from such organisations. Leaders of the Fitzroy Valley did not want to waste any time doing research that was not going to directly benefit the community. This is echoed in the literature by other Indigenous communities [3, 4, 7, 41–56].

The importance of showing respect to Aboriginal people was a recurring theme throughout the interviews. There is still a lack of acknowledgement by non-Indigenous researchers of the cultural hierarchy that is deemed with reverence by Aboriginal community leaders. Western facilities and processes fail to attribute any power to cultural elders to reflect their social standing within the community. Even body language, including eye contact, illustrates a difference in approach between Indigenous and non-Indigenous people. There are often assumptions as to who is important in communities. One Aboriginal community leader described that an elder may be sitting on the ground at the entrance to a community, while Western researchers are looking for a man in a suit that can speak English. Leaders said that the elders are the keepers of the knowledge. It may be unclear as to what this knowledge is, but if elders are to be engaged in research as holders of knowledge, it is imperative that they are addressed with respect. Local Community Navigators can provide guidance in how to dress, who to talk to and how to approach people. There are guidelines for some places in Australia which provide specific practical instructions for engaging with Aboriginal communities that are in-line with much of the advice provided by community leaders during our project [4, 58, 59]. Although there are a number of commonalities between communities, it is important not to generalise from one Indigenous community to another as they each have their own unique cultural protocols [4].

Aboriginal community leaders should be able to choose to be interviewed at a time and place where they feel most comfortable. In the interview setting, Aboriginal community leaders often chose to be interviewed in a place where a family member could witness the proceedings from a distance. Respect for working on country was also raised as in issue by Aboriginal community leaders. They frequently talked about the river, the desert, the seasons and hunting through their stories and responses, exemplifying the importance to them of the land and nature. The value of working on country is supported by other researchers [54].

Aboriginal community leaders also discussed that it was important for researchers to have respect for Aboriginal people’s time and to be flexible. The importance of a flexible approach when working with Aboriginal people is highlighted in the research setting [4, 53] and the clinical setting [55]. Leaders of Aboriginal communities frequently operate in both the Aboriginal and the Western world and are often over-committed to a number of responsibilities. They only have a small window of time in their week to volunteer to participate in research. In our study it was appreciated when researchers were flexible in their schedule so that community leaders could participate at a time that suited them most. It was also appreciated when researchers spent time to build trusting relationships with the wider Aboriginal community prior to seeking consent. This is highlighted as important by Aboriginal communities in Canada [56]. When talking about research, potential participants may need time to go to another community to talk to a senior for advice. ‘Lore’ or ‘sorry business’ may also interrupt the research process and such cultural protocols need to be respected. This may add pressure to the budgeting and the timeline of projects especially if they are supported by a grant and have a prior-approved deadline. Researchers may be tempted to try and rush their project and this was specifically mentioned as something to avoid by a number of Aboriginal community leaders.

One method of approaching research is similar to the ‘social yarning’ that is described by Dawn Bessarab [27]. This is a casual introduction prior to the formal ‘research topic yarning’ [27].

Most leaders agreed that continuous communication and true partnership between researchers and communities would prevent problems from occurring. The progress of the research project would be facilitated with the supportive guidance of the local Aboriginal research team members. One Aboriginal community leader said it was imperative that a local Aboriginal person is brought along even for participants who could speak English. This allows the researcher to be introduced by someone familiar and trusted in the community and who is available to address any further questions or concerns. In the Fitzroy Valley, only certain people are permitted to work with certain language groups and entre certain communities. This concept is common in other Aboriginal communities of Australia [60]. The need for an interpreter is supported by other research, however it is noted that even with someone present to interpret, it can be difficult to explain complex research concepts and scientific jargon across two languages and two cultures [3, 4, 6, 57, 60].

Through working together with local interpreters or Community Navigators, there is a two way learning process. This may also be considered as research capacity building for local Aboriginal researchers. Their involvement should be acknowledged through payment, authorship or co-presentations. At the outset of a project ownership of data needs to be addressed and all data collected should be available to the relevant community. On the other side, the non-Aboriginal researchers should receive cultural capacity building when working with Aboriginal researchers. As indicated in this project, there is much to learn about protocols for engaging elders; which language group to talk to; when it is OK to talk to someone after ‘sorry business’ etc. All of this should be guided through the local Aboriginal researcher or Community Navigator. In this way the likelihood of breaking cultural sanctions or causing upset can be minimised. There is an unwritten rule that research should not take priority over more pressing issues that impact participant’s lives and this might mean research protocols need to be amended, possibly to the point of seeking ethics approval for such changes [4].

Obtaining community consent may be a long process. As one community leader explained, consent doesn’t end at the beginning of the project with the signing of an agreement. The process of seeking consent continues throughout a project. It is imperative to maintain open communication in order to address any issues that arise early, and to produce outcomes that are valuable to the community, in a way that is culturally respectful [4]. Community consent needs to be obtained and maintained before one can approach individuals for consent [3, 4, 57]. It is also important to have a means of evaluating understanding of the research process as this is rarely reported as shown in our international systematic review [3]. In this way free, prior, informed consent may be sought for research with remote Aboriginal communities.

### Strengths and limitations of this study

The Picture Talk Project demonstrates strength through its collaborative design [4, 23]. This project was invited by Aboriginal community leaders of the Fitzroy Valley who were involved throughout the project. Their contribution is acknowledged as chief investigators and co-authors. The project was designed together from start to end. Local Aboriginal people were employed as Community Navigators onto the research team and interpreted the research into local Kimberley languages and provided cultural guidance for non-Aboriginal team members. This process was applauded as being culturally respectful through direct feedback from participants during interviews and indirectly through community members. In this qualitative study saturation of themes was achieved. Many of the leaders of community organisations in the Fitzroy Valley participated in this study and there was a spread of leaders from each of the dominant language groups, from a variety of community organisations and of different gender and age groupings. Thematic analysis of interview transcripts was conducted in a scientifically rigorous way using an integrated approach with grounded theory with line-by-line coding in an iterative process [24–26, 29, 36–39]. This was facilitated by NVivo10 qualitative software [35, 36].

A limitation of this research is that the majority of the analysis of interview transcripts was conducted through the eyes of researchers who are not local to the Fitzroy Valley community and do not identify as Aboriginal. It is impossible to avoid this impacting on the way in which interviews were coded and results were interpreted. To address this issue, sample extracts of data were coded by local Aboriginal Community Navigators and final results approved by Aboriginal community leaders in order to validate coding. It would be preferable to have all interviews conducted and analysed by a Community Navigator who spoke a local language but this was not possible due to limited time and resources. However, some interviews were conducted in a local language and interpreted into English by Community Navigators. Certain nuances of the original language may have been missed through this process.

## Conclusion

The Picture Talk Project is the first of its kind and provides important messages for researchers worldwide. In this study Australian Aboriginal leaders from remote communities were interviewed specifically about their experiences with research, community engagement and consent for research. They recommend that researchers give value to the knowledge and research processes that already exist within Aboriginal communities. They emphasise that research should only be conducted if it will provide benefits to the community involved. Researchers who are not local to the area must be respectful of Aboriginal people and the cultural hierarchy within the community. They should recognise the value of working on country. Non-Aboriginal researchers need to be particularly sensitive to the competing cultural priorities of Aboriginal community leaders and the community and be prepared to be flexible with time. Time needs to be allowed for consideration of research participation, project design and conduct of the project. There needs to be strong and continuous communication between the research team and participating communities. Research should be conducted in the language of preference of the participants and in a way that enables them to understand the project. Understanding of the consent process should be evaluated. It is imperative that local people are involved in the design and conduct of research, that results are communicated back to the community in a timely and understandable way and that the community feels ownership of the data. Before research can begin it is important to seek community approval and spend time forming trusting relationships with the community. Following this, the conversation about the individual consent process for participants can begin. Only if this process is followed will we ensure free, prior, informed consent for research and conduct research with Aboriginal communities in the most respectful way.

The main message from Aboriginal community leaders of The Picture Talk Project is that research teams must include respected and knowledgeable representatives from Aboriginal communities in the leadership and design of a research project from the start. Knowledge of cultural protocols is essential but these might take years to acquire so a researcher should always have a respected local person to help them negotiate these. This approach should be integral to any research that engages community-based Indigenous people. Protocols that will improve the governance, partnerships, and ethical guidelines of future research projects are needed. Stronger research outcomes will result when the whole process embodies culturally-meaningful respect for local Indigenous people and their understandings in every step of the research journey.

## Additional file

Additional file 1: Interview questions designed in collaboration with Aboriginal leaders on the research team. These provided a guide for the interviews, however interviews were semi-structured, with the question order depending on the participant responses. (DOCX 676 kb)

## Abbreviations

AK: Anastasia Kindrakewich
AM: Associate Professor Alexandra Martiniuk
BMedSci(Hons): Bachelor of Medical Science with Honours
CEO: Chief Executive Officer
COREQ: Criteria for reporting qualitative research
EE: Professor Elizabeth Elliott
EF: Dr Emily Fitzpatrick
FASD: Fetal Alcohol Spectrum Disorder
HD’A: Ms Heather D’Antoine
JO: Ms June Oscar
KALACC: Kimberley Aboriginal Law and Culture Centre
MBBS: Bachelor of Medicine and Bachelor of Surgery
MC: Ms Maureen Carter
NHMRC: National Health and Medical Research Council
P: Participant
PB: Mr Percy Bulagardie
SE: Ms Sharon Eadie
TL: Mr Tom Lawford
