# Supplementary material for: The Picture Talk Project: Starting a Conversation with Community Leaders on Research with Remote Aboriginal Communities of Australia
Source: BMC Med Ethics. 2017 May 11;18:34. doi: 10.1186/s12910-017-0191-z (PMC5426070; doi:10.1186/s12910-017-0191-z)
Supplement: Additional file 1: — Interview questions designed in collaboration with Aboriginal leaders on the research team. These provided a guide for the interviews, however interviews were semi-structured, with the question order depending on the participant responses. (DOCX 676 kb) [file 12910_2017_191_MOESM1_ESM.docx]

**INTERVIEW TOPIC GUIDE**

1. Interview Preparation

- Ensure consent and demographics form filled in and language preference noted
- Check recording device/ batteries / room availability
- Quiet setting, ensure interviewee is comfortable
- Explain the purpose of the interview
- Address terms of confidentiality (who has access to answers and analysis)
- Explain the format of the interview – the type of interview and its nature
- Indicate how long the interview usually takes
- Ask if there are any questions before the interview starts
- Ask permission to record the interview

1. Start interview

- Note the time, date, location and attendees of interview
- Thank participant for taking the time to do this interview
- Use standardised open ended topic questions

(Open ended, neutral and clear questions)

1. Close interview

- Check if participant has any other information to add
- Thank participant for their time and note the end of the interview.
- Write any observational notes immediately from interview experience.
- Add to any notes taken within interview.

**INTERVIEW QUESTIONS**

1. How do you think permission should be granted for research with the Aboriginal communities of the Fitzroy Valley?
2. What do you think about the way research projects approach this process?
3. Does this process respect Aboriginal culture?
4. What do researchers need to know when consulting communities and their leaders to see if they can do a project?
5. How do researchers approach a community?
6. How do they start communication with the community leader?
7. What steps need to be taken to ask permission from a community leader?
8. Is permission from the community leader enough to start a project or does permission need be sought from the community? If so, how is this done?
9. Who do researchers approach first for the Fitzroy Valley?
10. Are there any organisations that need to be involved?
11. Which leaders do they need to talk to?
12. Do they have to talk to all the leaders for every project?
13. How much information should a project give when talking about a proposed research project?
14. How should this information be delivered?
15. Can you tell me from your experience an example of a research project that worked well with the Fitzroy Valley Communities? What made it a good?
16. Can you tell me from your experience an example of a research project that did not work well with the Fitzroy Valley Communities? What was not so good?
17. If permission is granted for a project, how long does the permission last?
18. Do researchers need to “check in” that the leaders are happy with the project? How often? How do they do this?
19. Do you have any things you think researchers should NOT do?
20. Is there any other information you would like to share?
